# Application of Pulsed Electric Field During Malting: Impact on *Fusarium* Species Growth and Mycotoxin Production

**DOI:** 10.3390/toxins16120537

**Published:** 2024-12-12

**Authors:** Nela Prusova, Marcel Karabin, Lukas Jelinek, Jana Chrpova, Jaroslava Ovesna, Pavel Svoboda, Tereza Dolezalova, Adam Behner, Jana Hajslova, Milena Stranska

**Affiliations:** 1 Department of Food Analysis and Nutrition, University of Chemistry and Technology, Prague, Technicka 3, 166 28 Prague, Czech Republic; nela.prusova@vscht.cz (N.P.); dolezaat@vscht.cz (T.D.); adam.behner@vscht.cz (A.B.); jana.hajslova@vscht.cz (J.H.); 2 Department of Biotechnology, University of Chemistry and Technology, Prague, Technicka 5, 166 28 Prague, Czech Republic; marcel.karabin@vscht.cz (M.K.); lukas.jelinek@vscht.cz (L.J.); 3 Crop Research Institute in Prague, Drnovska 507/73, 161 06 Prague, Czech Republic; chrpova@vurv.cz (J.C.); ovesna@vurv.cz (J.O.); pavel.svoboda@vurv.cz (P.S.)

**Keywords:** pulsed electric field, mycotoxins, *Fusarium* micromycetes, U-HPLC-HRMS/MS, RT-PCR, malting

## Abstract

The increasing contamination of cereals by micromycetes and mycotoxins during malting still poses an unresolved food safety problem. This study characterises the potential of the novel, rapidly developing food production technology of Pulsed Electric Field (PEF) to reduce the viability of *Fusarium* fungi and the production of mycotoxins during malting. Barley, artificially inoculated with four *Fusarium* species, was treated by PEF with two different intensities and then malted using a standard Pilsner-type technology. Concentrations of fungi were quantified by RT-PCR, expression of fungal growth-related genes was assessed using mRNA sequencing, and mycotoxin levels were analysed by U-HPLC-HRMS/MS. Despite the different trends for micromycetes and mycotoxins after application of variously intense PEF conditions, significant reductions were generally observed. The greatest decrease was for *F. sporotrichioides* and *F. poae*, where up to six fold lower levels were achieved for malts produced from the PEF-treated barley when compared to the control. For *F. culmorum* and *F. graminearum*, up to a two-fold reduction in the PEF-generated malts was observed. These reductions mostly correlated with a decrease in relevant mycotoxins, specifically type A trichothecenes.

## 1. Introduction

Mycotoxins, toxic secondary metabolites produced by various microscopic filamentous fungi, pose significant risks to human and animal health due to their toxic properties [1,2]. Among the various fungal species associated with mycotoxin contamination, *Fusarium* pathogens have emerged as a major concern in agricultural cultivation of small grain cereals and further processing. *Fusarium* species and their mycotoxins usually contaminate grains at the pre-harvest stage, and their levels can also increase during inappropriate storage conditions [3,4]. Some food processing technologies, specifically those involving soaking of grains, germination, and increased enzymatic activity (like malting), are also susceptible to the spread of micromycete pathogens and the formation of mycotoxins [5,6,7]. During malting, development of fungal contamination, accompanied by an increase in mycotoxins, occurs mainly after barley steeping during germination, as favourable conditions (temperature around 14–18 °C and 95–100% air humidity) support fungal development [8]. After malting, some mycotoxins, especially the polar ones such as trichothecenes (deoxynivalenol (DON) and its modified form deoxynivalenol-3-glucoside (D3G), nivalenol (NIV), HT-2 (HT-2) and T-2 (T-2) toxins) and *Alternaria* mycotoxins (notably tenuazonic acid (TEA) and tentoxin (TEN)), are easily transferred to the final beers and may pose a risk to consumers [5,7,9].

Since fungal/mycotoxin contamination is a problem, both in raw materials as well as processed foods, strategies to minimise their occurrence at different stages of the production chain are of great importance. Currently, there are several physical-, chemical-, and biological-based strategies that can be applied during cereal growth and processing. Pre-harvest strategies focus on preventing mycotoxin formation by inhibiting fungal growth and include maintaining good agricultural practices, using fungicides, or selecting resistant crop varieties. However, post-harvest reduction of micromycetes and mycotoxins is generally very difficult because once contamination is present, it is virtually impossible to remove it. Several strategies have recently been investigated for minimisation of *Fusarium* fungi, including cold plasma [10], pulsed light [11], and ozone treatment [12]. Among these, the pulsed electric field (PEF), which uses high-voltage electric pulses of very short duration to cause disruption of biological membranes and cell death, shows strong potential to affect fungal mycelia and spores and reduce fungal viability [13,14,15,16,17,18]. Higher PEF energies have also been shown to disrupt/modify mycotoxin structures, which may be promising from the viewpoint of mycotoxin degradation [17]. Considering the fact that PEF treatment can only be applied to liquid foods or within food technologies using ‘wet’ technological steps (e.g., washing or steeping, as the electric field needs a current conductor), its use within malting seems to be very logical and easy to perform.

The aim of this study was to characterise the fate of most common *Fusarium* pathogens of barley (in particular, *F. culmorum*, *F. graminearum*, *F. sporototrichioides*, and *F. poae*) and types of mycotoxins produced (type B trichothecenes, type A trichothecenes, zearalenone (ZEA), and enniatins) during malting technology after initial PEF intervention. To the best of our knowledge, this is the first study describing the behaviour of these microbial and chemical contaminants during malting after PEF treatment of input barley, which may significantly contribute to the future application of PEF technology in the malting industry.

## 2. Results and Discussion

### 2.1. Input Material

The PEF treatment and malting experiments were performed with Bojos barley inoculated with strains of four *Fusarium* species (*F. culmorum*, *F. graminearum*, *F. sporotrichioides*, and *F. poae*), the most common micromycetes occurring in cereals of the temperate climatic zone. In this study, two variously intense PEF treatments of barley, followed by malting of this barley, were carried out in two consecutive years, 2021 and 2022 (due to the limited shelf-life of the barley to be used for malting, the fresh material had to be grown for the second malting experiment; otherwise, the technological parameters of the ‘old’ barley, specifically the germination energy, could be negatively affected). It should be clarified that the reason for the one-year time gap between the malting trials was that these were the final verification of extensive optimisation studies taking place during the two-year period, where the intensity of the PEF treatment was optimised depending on germination ability and enzymatic activity of germinated barley grains. The optimisation experiments as such are discussed in another separate article [18], and here we present detailed information on the fate of *Fusarium* micromycetes and their mycotoxins during malting of the PEF-treated barley.

As concerns the content of mycotoxins present in the input material, in both barley batches, type B trichothecenes (DON and D3G), type A trichothecenes (HT-2 and T-2), and enniatins (enniatin B (Enn-B) and enniatin B1 (Enn-B1)) were the most abundant (Table 1). Surprisingly, despite the considerably lower concentration of *Fusarium* fungi in 2021 (on average, approx. four times lower than in 2020; see Table 1), comparable or even higher concentrations of mycotoxins were observed. The phenomenon of poor correlation between the levels of microscopic fungi and mycotoxins is very common because toxin production depends on environmental factors such as temperature and humidity, as was recently highlighted in a very complex 16-year study assessing fungi and mycotoxins in Swedish cereals [19]. The spectrum of mycotoxins present in barley more or less referred to the toxigenic potential of the fungal strains used for inoculation (see the results of laboratory cultivation of all *Fusarium* strains and their chemotypes in Table 2). The slight differences in mycotoxin representation can be explained by differences in gene expression, which is very environmentally dependent, and by interspecies competition with other natural micromycetes in the field [20,21,22].

### 2.2. PEF Treatment of Barley and Subsequent Malting

**Figure 1:**
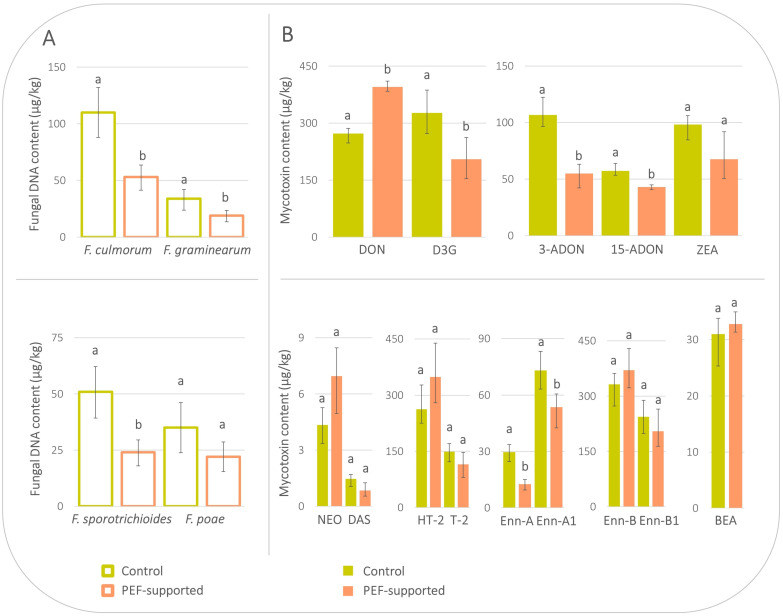
Levels of *Fusarium* species (**A**) and relevant mycotoxins (**B**) in final malts produced from pre-soaked barley without PEF treatment (control) and pre-soaked barley treated by PEF (*experiment I*). Error bars represent the variability between individual averaged samples; *n* = 9. Data were statistically processed using a two-sample *t*-test with unequal variance; statistical differences (*p*-value < 0.05) are indicated by letters. If the result was not statistically significantly different between the control and PEF-supported sample, both columns are marked with the letter ‘a’. In the case of a statistically significant difference, the PEF-supported sample is marked with the letter ‘b’.

As explained in the paragraph above, the PEF conditions of *experiment I* (performed in 2021) were chosen on the basis of our previous extensive optimisation experiment [18], in which various PEF parameters (in particular the electric field strength, number of pulses, pulse width, but also the pre-soaking period prior to PEF intervention) were assessed in relation to the germination potential of the grains. The germination test of PEF-treated barley grains using Petri dishes was performed in that study, and the most intensive PEF conditions that did not negatively affect the germination ability (in particular 3 h 30 min water pre-soaking period and the PEF treatment of 6.0 kV/cm, 100A, 100 bipolar pulses, and 5 µs pulse width) were used for the treatment of one kilogram of barley intended for malting in our study (see Section 4.2). The results of fungal DNA and mycotoxin concentrations in the final malts are shown in Figure 1. In the figure, *Fusarium* species and specific mycotoxins produced by them are depicted side by side. As can be seen in this figure, a reduction in the fungal DNA content of all four *Fusarium* species (*F. culmorum*, *F. graminearum*, *F. sporotrichioides*, and *F. poae*) in the PEF-supported malt could be observed. The concentrations of fungal DNA in PEF-supported vs. control malt were 53 vs. 110 µg/kg for *F. culmorum*, 19 vs. 34 µg/kg for *F. graminearum*, 24 vs. 51 µg/kg for *F. sporotrichioides*, and 22 vs. 35 µg/kg for *F. poae*, respectively. These results are in good agreement with other studies where *Fusarium* species reduction has also been demonstrated, specifically *F. graminearum* in seeds of different crops [13] and *F. oxysporum* in nutrient solutions [14,15]. In the study of Palicova et al., viability of spores of the same *Fusarium* species (*F. culmorum*, *F. graminearum*, *F. sporotrichioides*, and *F. poae*) was proven to be decreased after application of PEF energy to spore suspensions [16]. Similarly, a statistically significant decrease in the majority of mycotoxins was also observed, which confirms the positive effect of PEF on both *Fusarium* fungi and mycotoxins [16,17,18,19,20,21]. However, as shown by the determination of activity/concentration of key enzymes in this final malt, which play essential roles in subsequent beer brewing, the PEF conditions used in *experiment I* have been proven to be too intensive. The enzymatic activity of α-amylase, β-glucanase, and diastatic power was approx. one-half in PEF-supported malt (66 U/kg, 80 U/kg, and 202 WK u., respectively) compared to control malt (240 U/kg, 603 U/kg, and 364 WK u., respectively) (Table 3). The enzymes occur in the endosperm of barley grains, so we could hypothesise that the 3 h 30 min pre-soaking period was too long and the water content enabled too deep penetration of the electric pulses and subsequent enzyme inactivation. As was previously described by another group, PEF energy is able to change the secondary, tertiary, and quaternary structures of proteins, thus affecting the function of enzymes by destroying their active sites and substrate tunnels [23].

**Figure 2:**
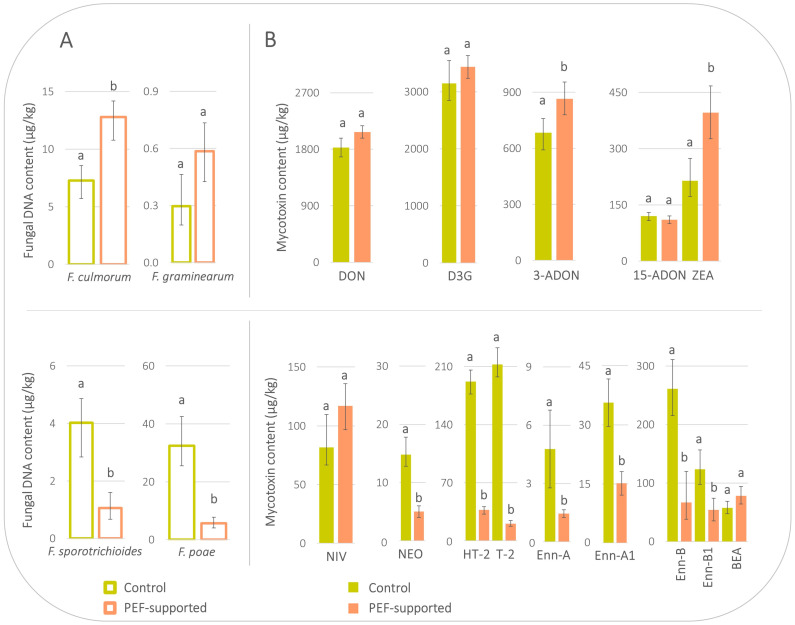
Levels of *Fusarium* species (**A**) and relevant mycotoxins (**B**) in final malts produced from pre-soaked barley without PEF treatment (control) and pre-soaked barley treated by PEF (*experiment II*). Error bars represent the variability between individual averaged samples; *n* = 9. Data were statistically processed using a two-sample *t*-test with unequal variance; statistical differences (*p*-value < 0.05) are indicated by letters. If the result was not statistically significantly different between the control and PEF-supported sample, both columns are marked with the letter ‘a’. In the case of a statistically significant difference, the PEF-supported sample is marked with the letter ‘b’.

Therefore, further optimisation of PEF had to be carried out [18], resulting in *experiment II* (performed in 2022), with a reduced pre-soaking period (10 min instead of 3 h 30 min), but higher conductivity of electrolyte (phosphate buffer instead of water) and higher overall energy per sample mass (152 J/g vs. 30 J/g, see Section 4.2). According to current knowledge, *Fusarium* micromycetes and mycotoxins are primarily present on the grain surface and in the covering layer of the grains [24,25,26], so a shorter pre-soaking period was presumed to be effective for pathogen destruction but safer for enzymes. And indeed, this time the enzymatic activities of α-amylase, β-glucanase, and diastatic power were fully comparable for both PEF-supported (203 U/kg, 503 U/kg, and 255 WK u., respectively) and control malt (211 U/kg, 448 U/kg, and 251 WK u., respectively) (Table 3). In other words, as the technological quality was not negatively influenced by PEF, the PEF-induced changes in the fate of *Fusarium* fungi and mycotoxins during malting could be investigated in more detail. From Figure 2, it is evident that *experiment II* had a statistically significant positive effect in reducing the levels of *F. sporotrichioides* and *F. poae* in the final malts (concentrations in PEF-supported vs. control malts were 1 vs. 4 µg/kg and 6 vs. 32 µg/kg, respectively). Similarly, statistically significant PEF-induced reductions were observed for relevant mycotoxins, namely NEO (5 vs. 15 µg/kg), HT-2 (37 vs. 192 µg/kg), and T-2 (21 vs. 213 µg/kg), followed by enniatins, Enn-A (1 vs. 5 µg/kg), Enn-A1 (15 vs. 36 µg/kg), Enn-B (67 vs. 261 µg/kg), and Enn-B1 (54 vs. 124 µg/kg). On the other hand, levels of *F. culmorum* and *F. graminearum* behaved differently and, surprisingly, were increased in the PEF-supported malt (content in PEF-supported vs. control malt was 13 vs. 7 µg/kg and 0.6 vs. 0.3 µg/kg, respectively), despite the fact that the PEF energy was higher than in the previous case. However, it should be noted that specifically for *F. graminearum*, the absolute concentration of fungal DNA was very low, and its increase after the PEF intervention was not statistically significant. As for mycotoxins, although the increased presence of *F. culmorum* corresponded to higher levels of relevant mycotoxins in the PEF-supported malt when compared to control (specifically for DON and its modified forms and for ZEA), these differences were slight and not statistically significant for most of them (except for ZEA, where the concentration was approx. twice in the PEF-treated malt when compared to the control).

**Figure 3:**
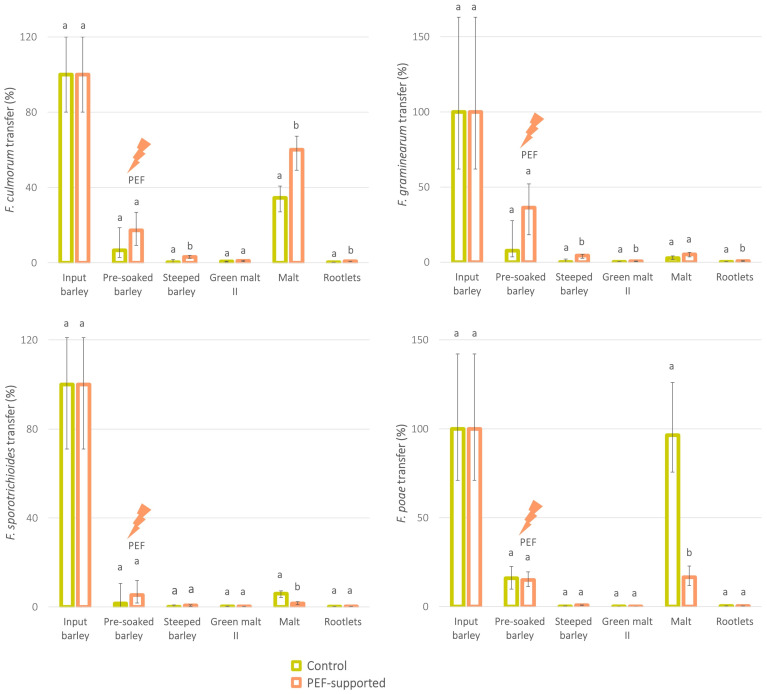
Transfer of micromycetes in the dry matter of intermediates during the production of malt from pre-soaked barley without PEF treatment (control) and pre-soaked barley treated with PEF (*experiment II*). The total amount of each micromycete in the input barley = 100%. Error bars express the variability between individual averaged samples (*n* = 9). Data were statistically processed using a two-sample *t*-test with unequal variance; statistical differences (*p*-value < 0.05) are indicated by letters. If the result was not statistically significantly different between the control and PEF-supported sample, both columns are marked with the letter ‘a’. In the case of a statistically significant difference, the PEF-supported sample is marked with the letter ‘b’. The results for the ‘Green malt I’ intermediates were not included due to visible mould contamination and outlying values in these samples.

Looking at the detailed balance of micromycetes during malting (Figure 3), it is evident that during barley steeping, the *Fusarium* DNA for all species was significantly reduced by rinsing (see decrease of *Fusarium* DNA from ‘pre-soaked barley’ to ‘steeped barley’). During the next stage, germination, these levels remained about the same, with minimal or no differences between the PEF-treated and control variants (see ‘steeped barley’ and ‘green malt II’). The most significant changes in *Fusarium* pathogen levels were apparently observed during kilning of the green malt (see the *Fusarium* DNA increase from the ‘green malt II’ to the ‘final malt’). Unfortunately, we did not perform more detailed sampling of the kilning process, so we are not able to describe this critical phase of the technology in more detail. Nevertheless, this observation is in good agreement with another previously published study where significant increases in mycotoxin levels during the early kilning phases, associated with the development of fungal infection at temperatures of around 45 °C, were observed [7]. As already mentioned above, contrary to the *F. sporotrichioides* and *F. poae*, micromycetes *F. culmorum* and *F. graminearum* showed higher concentration levels of fungal DNA in PEF-supported malt when compared to the control. It is however important to emphasise that despite this fact, the total level of pathogen DNA did not exceed the initial levels in the input barley, dismissing the negative effect of PEF technology. In reality, the process of fungal infection and interspecies competition is a very complex procedure that depends on various factors, including penetration depth of the pathogen into the grain, moisture of substrate during anthesis, natural crevices within the spikelet [27], or the particular species of the cereals [28]. Differences in pathogenicity between particular *Fusarium* species and their competitiveness during multi-species fungal development [4,29,30] are probably even more essential. Synergistic or competitive interactions between fungi is a well-known phenomenon [21], and numerous studies presenting *F. graminearum* and *F. culmorum* as the most competitive *Fusarium* species [21,31,32,33] finally support the theory that the rather high PEF energy focused on the surface grain layers suppressed *F. poae*/*F. sporotrichioides* in favour of *F. culmorum*/*F. graminearum* growth.

These results were finally confirmed also by the transcriptomic analyses, which are summarised in Table 4 and Table S1 of the Supplementary Material. The differentially expressed genes of both *F. culmorum* and *F. graminearum* upregulated after the PEF treatment referred to cell wall phiA protein associated with cell wall morphogenesis [34]. Other genes upregulated after the PEF treatment in *F. culmorum* encoded lysophospholipase, previously described to facilitate penetration of fungi into the host [35], and alcohol oxidase, which metabolises plant aromatic alcohols, supporting colonisation of host tissues [36]. In contrast, for *F. poae*, we observed downregulation of genes encoding proteins with high similarity to ATP synthase, an enzyme positively correlated with *Fusarium* virulence [37], and endo-1,4-beta-xylanase C, a cell wall-degrading enzyme contributing to fungal spread [38]. These results are consistent with our DNA assays. The only gene of *F. poae* that was upregulated in PEF-supported samples encoded a protein with high similarity to cytochrome P450 monooxygenase. This enzyme is known for its roles in ergosterol synthesis and virulence [39], as well as in reactive oxygen species accumulation and cell death [40], which aligns with our findings. Regarding *F. sporotrichioides*, we observed downregulation of a gene encoding a protein with high identity to the Ecp2 effector protein. Ecp2 effector proteins are extracellular fungal proteins contributing to virulence [41], so their decreased expression in PEF-treated samples corresponds with the reduced DNA of *F. sporotrichioides* found in our samples.

**Figure 4:**
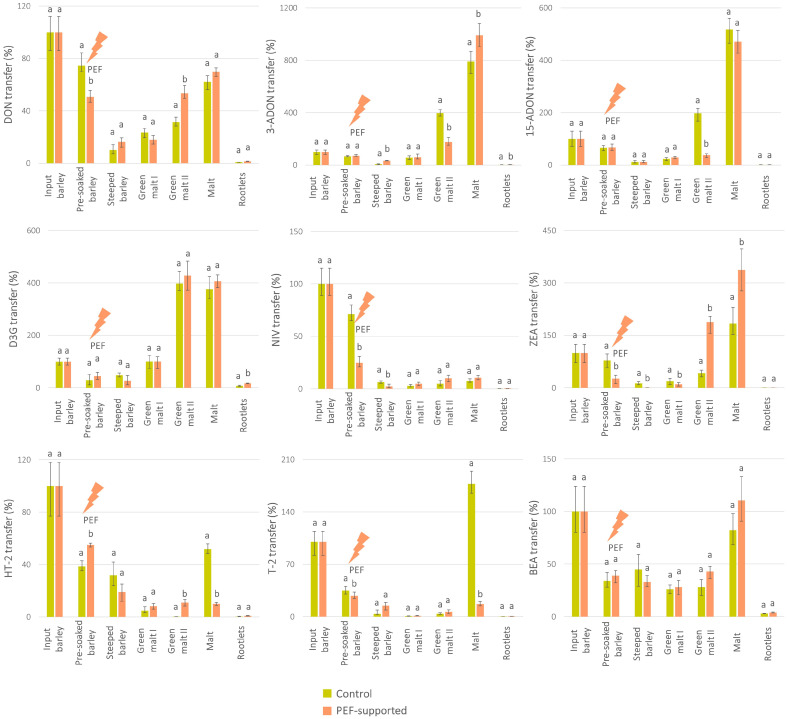
Transfer of mycotoxins produced by *Fusarium* species used for barley inoculation into the dry matter of intermediates during the production of malt from pre-soaked barley without PEF treatment (control) and pre-soaked barley treated with PEF (during *experiment II*). The total amount of each mycotoxin in the input barley = 100%. Error bars express the variability between individual averaged samples (*n* = 9). Data were statistically processed using the two-sample *t*-test with unequal variance; the statistical differences (*p*-value < 0.05) are indicated by letters. If the result was not statistically significantly different between the control and PEF-supported sample, both columns are marked with the letter ‘a’. In the case of a statistically significant difference, the PEF-supported sample is marked with the letter ‘b’.

The balance graph of mycotoxins in malting intermediates (Figure 4) shows interesting trends, specifically for DON and its derivatives 3-ADON and 15-ADON as mycotoxins relevant to the *F. culmorum* chemotype used in this study. Contrary to the behaviour of the fungal producer, the most pronounced increase in mycotoxins was already observed during germination, in particular in the ‘green malt II’ intermediate. In other words, an increase in these mycotoxins was observed in the technological step, where the content of fungal DNA was still low. This trend, which was rather independent of the PEF treatment, is a good illustration of the previously described asymptomatic infection phenomenon [42,43].

On the other hand, a strong and statistically significant reduction in PEF-supported malts when compared to the control was observed for type A trichothecenes relevant to *F. sporotrichioides* and *F. poae* chemotypes, namely NEO, HT-2, and T-2. In this case, the behavioural trends of HT-2 and T-2 during the malting process (depicted in Figure 4) follow the patterns of their producers (shown in Figure 3), including the increase in mycotoxins concentrations from the ‘green malt II’ to the ‘final malt’. For NEO, the concentrations in malting intermediates were below the quantification limits, so the balance graph could not be shown. The PEF-induced reduction of HT-2 and T-2, the most toxic trichothecenes occurring in cereals [44], highlights the great impact of PEF on the safety of malt and malt-based foods. This positive outcome is most probably associated with the decreased viability of the *F. sporotrichioides* and *F. poae* pathogens, as the fungal cells and spores are damaged through electroporation, a typical consequence of PEF treatment on microorganisms [16,45]. The direct effect of PEF on mycotoxin molecules (degradation/transformation), which was demonstrated recently by Stranska et al. [17], can be considered for the first intermediate sample only (i.e., ‘pre-soaked barley’ treated by PEF), since for subsequent samples, the influence of external factors such as temperature, humidity, and others affects fungal growth as well as mycotoxins. Indeed, when comparing the T-2/HT-2 concentrations in the PEF-treated ‘pre-soaked barley’, we can observe a decreased concentration of T-2, accompanied by an increased concentration of HT-2, which is probably associated with PEF-induced T-2 deacetylation.

## 3. Conclusions

This study focused on describing the effect of PEF on the reduction of *Fusarium* species and minimisation of mycotoxins during malting. The most significant results relevant to the practical impact of PEF on the final malt are summarised below:

- The PEF treatment significantly reduced most of the *Fusarium* species investigated in this study (i.e., *F. culmorum*, *F. graminearum*, *F. sporotrichioides,* and *F. poae*), where the extent of the reduction depended on the particular PEF parameters and conditions. Recorded up-/down-regulation of genes associated with fungal growth confirmed these results at the transcriptomics level.
- The reduction in *Fusarium* species was mostly associated with a reduction in the levels of relevant mycotoxins, specifically for *F. sporotrichioides* and *F. poae* and type A trichothecenes, T-2, and HT-2 toxins. The levels of DON derivatives and ZEA produced by *F. culmorum* and *F. graminearum* were more condition-dependent; specifically for ZEA, a statistically significant increase was observed under the technologically relevant PEF conditions.
- Despite the fact that verification of the presented results would be needed in up-scaled conditions of real malting houses, this study indicates the great potential for PEF in the fight with the ubiquitous problem of mycotoxin contamination of malt. The results of this study represent important pilot data, revealing opportunities for further follow-up research.

## 4. Materials and Methods

### 4.1. Input Material Production and Characterisation

Artificially inoculated barley of cultivar Bojos, which is most commonly used for the production of Pilsner-type malt, was used as input material for the PEF experiments and follow-up malting, performed in two consecutive years (2021 and 2022). In both cases, a highly pathogenic strain of *F. culmorum*, three strains of *F. graminearum*, two strains of *F. poae*, and three strains of *F. sporotrichioides* were used for inoculation. The production procedure was performed in the trial fields of the Crop Research Institute in Prague, according to Chrpova et al. [46]. After grain harvest, the particular *Fusarium* species and mycotoxins were quantified as described in Section 4.3 and Section 4.5, respectively. For the follow-up malting experiments, two one-kilogram batches were prepared from each barley harvest and processed as described in Section 4.2.

In order to verify the toxigenic potential of *Fusarium* strains used for barley inoculation, analysis of *Fusarium* mycotoxins was performed. *Fusarium* isolates deposited in the Culture Collection of Microorganisms of Crop Research Institute in Prague were cultivated on Petri dishes containing potato dextrose agar (PDA, HiMedia Laboratories, Czech Republic) at 20 °C in the dark. After seven days, the discs (diameter 5 mm) were cut from the dishes with fungal mycelium, put on the new Petri dishes with PDA, and cultivated at 20 °C in the dark for 21 days. Each Petri dish, containing a specific *Fusarium* strain, was prepared in triplicate and then subjected to mycotoxin analysis according to Dzuman et al. [47] (see Section 4.5) with slight modifications. The entire content of each Petri dish was weighed and transferred to a 50 mL centrifuge tube, and the appropriate amount of acetonitrile was added, keeping the agar:acetonitrile ratio at 1:1 (*w*/*w*). The sample was then homogenised using a disperser (IKA, T 18 digital ULTRA-TURRAX^®^, Labortechnik, Germany), and the extraction was performed using a laboratory shaker for 30 min at 240 min^−1^ (IKA 240, IKA Labortechnik, Staufen, Germany). MgSO_4_ and NaCl were then added to maintain the ratios of acetonitrile:MgSO_4_ (10:4, *w/w*) and acetonitrile:NaCl (10:1, *w/w*). The sample was vigorously shaken by hand for 1 min, followed by centrifugation for 5 min at 10,000 min^−1^; 13,081× *g* (Rotina 380R, Hettich, Tuttlingen, Germany). The extract was placed in a vial and stored at –20 °C until analysis by U-HPLC-HRMS/MS, as described in Section 4.5.

Quantification of mycotoxins was performed by matrix-matched calibration by using the mycotoxin-free PDA agar (blank). Recoveries and repeatabilities expressed as relative standard deviations were checked by using the spikes at concentration levels of 10 and 100 μg/kg (*n* = 5), and were 70–110% and 0.5–5.7%, respectively.

### 4.2. PEF Treatment Conditions and Subsequent Malting

A PEF System (OMNIPEF, VITAVE, Czech Republic) with a cylindrical batch treatment chamber (7 cm i.d.) was employed for the experiments. Photographs of the PEF system and chamber are shown in Figure S1 of the Supplementary Material. The following conditions were set for each experimental variant:

*Experiment I*: One kilogram of barley was pre-soaked in tap water (electrolyte) for 3 h 30 min before PEF treatment in order to sufficiently wet the upper layers of the grains and assure effective permeation of the follow-up PEF energy to micromycetes. The PEF treatment conditions were as follows: voltage 6.0 kV/cm, current 100 A, number of bipolar pulses 100, and pulse width 5 µs. The total specific energy delivered to the barley was 30 J/g (calculated according to Equation (1)). In addition, one kilogram of control barley (without PEF treatment) was pre-soaked in tap water for the same time period and subjected to malting under the same conditions.

*Experiment II*: One kilogram of barley was pre-soaked for 10 min in 0.05 M phosphate buffer (ions of electrolyte allowed to achieve higher current during PEF treatment). After pre-soaking, the barley was exposed to PEF under the following conditions: voltage 3.8 kV/cm, current 200 A, number of bipolar pulses 100, and pulse width 20 µs. The total specific energy delivered to the barley was 152 J/g (calculated according to Equation (1)). In addition, one kilogram of control barley (without PEF treatment) was pre-soaked in 0.05 M phosphate buffer for the same time period and subjected to malting under the same conditions.

(1) $$Q=\frac{U\times I\times t\times n}{m}$$

Equation (1) was used to calculate the specific energy delivered in J/g for each PEF treatment, where ‘*Q*’ is the specific energy delivered (J/g), ‘*U*’ means the voltage (V), ‘*I*’ is the current (A), ‘*t*’ represents the length of 1 pulse (s), ‘*n*’ = the number of pulses multiplied by two (bipolar pulses), and ‘*m*’ is the weight of the total mass in the batch chamber (g). Amount of 20 g of pre-soaked barley was treated per run, resulting in approximately 100 repetitions of each experiment. The temperature increase caused by PEF was 1.5 °C for *experiment I* and 2.5 °C for *experiment II*.

**Figure 5:**
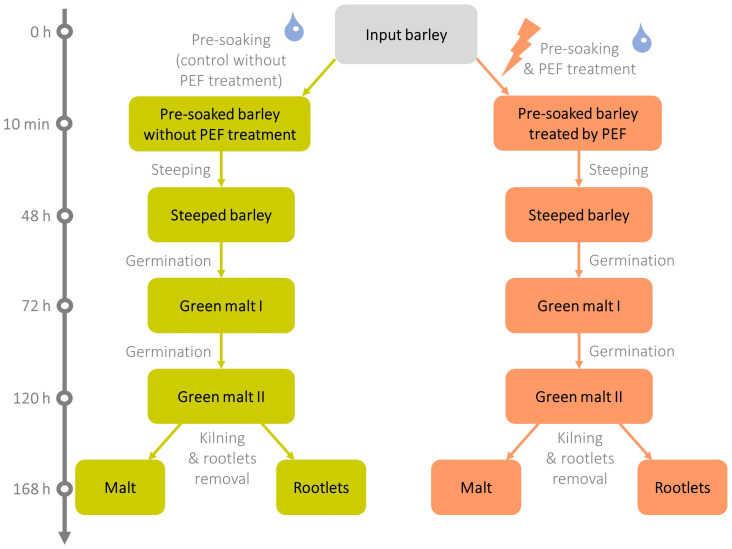
Timeline of malting experiment after the *experiment II* conditions. Malting intermediates analysed are shown in boxes.

Malting technology and sampling: Malting was conducted using an automatic micromalting system (RAVOZ^®^, Prague, Czech Republic), which comprises three separate modules for steeping, germination, and kilning. One kilogram of control barley and one kilogram of PEF-treated barley were used for malting. The processing of Pilsen-type malt involved the following steps: steeping for 48 h at 15 °C (8 h steeping, 12 h aeration, 8 h steeping, 12 h aeration, 4 h steeping, and 4 h dripping), germination for 72 h at 15 °C with 95–98% humidity, and kilning for 24 h using a drying air temperature gradient of 45–82 °C (45 °C for 6 h, 50 °C for 6 h, heating from 50 °C to 82 °C for 10 h and 82 °C for 2 h). In *experiment I*, approx. 100 g of input barley, control, and PEF-supported malt were collected for the DNA assay (Section 4.3) and mycotoxin analysis (Section 4.5) and frozen at −180 °C until respective analysis. In *experiment II*, approx. 100 g of input barley and each specific intermediate product (i.e., pre-soaked barley, steeped barley, green malt I, green malt II, malt, and rootlets) from control and PEF-treated malting were collected for analysis of DNA (Section 4.3) and mycotoxins (Section 4.5). The schematic of samples collected during malting is shown in Figure 5. For transcriptomics (Section 4.4), only the input barley and green malt II from PEF-treated and control variants were collected in amounts of 20 g. All samples collected were immediately snap-frozen in liquid nitrogen, homogenised using a cryogenic laboratory mill (IKA A11 basic, Verkon, Prague, Czech Republic), and then stored until further analysis (at −80 °C for DNA and mycotoxin analysis and at −180 °C for transcriptomics). The dry matter content of each sample was determined using a halogen moisture analyser (HR83, Mettler Toledo, Zurich, Switzerland) at 105 °C (results are shown in Table S2 in the Supplementary Material).

### 4.3. Analysis of Fusarium Species

DNA isolation was performed according to the method described by Ovesna et al. [48]. The DNA acquired from each sample replicate (*n* = 3) was subjected to an RT-PCR assay in three repetitions. RT-PCR reactions were performed in 25 μL volumes containing 12.5 μL of TaqMan^®^ 2x Universal PCR MasterMix (ThermoFisher Scientific, Waltham, MA, USA), 300 nM of both forward and reverse primers (Generi Biotech, Hradec Králové, Czech Republic), 200 nM of the probe (Generi Biotech, Czech Republic), 100 ng of template DNA, and nuclease-free water (Sigma, Steinheim, Germany). Real-time primers and probes for the quantification of *Fusarium* species DNA are provided in Table 5. The amplification regime included incubation at 50 °C for 2 min, followed by 10 min at 95 °C, 40 cycles at 95 °C for 15 s, and 60 °C for 60 s. Quantitative RT-PCR was conducted using the QuantStudio^TM^ 6 cycler (ThermoFisher Scientific, USA). Data for each dye reported were collected every 7 s from each well using the Design and Analysis software (ThermoFisher Scientific, USA), generating fluorescence profiles. The threshold cycle (Ct) was determined as the cycle number at which the fluorescence signal, indicating the exponential phase of PCR amplification, surpassed the background fluorescence. A dilution series of each *Fusarium* species DNA in the range of 0.9–1000 ng was used as standard in triplicate. Standard curves were constructed by correlating the known DNA concentrations with the Ct values obtained using the Sequence Detection System software (ThermoFisher Scientific, USA). Levels in unknown samples were determined by interpolating the measured Ct values using the regression equation derived from standard curves. The concentrations of DNA in samples differing in water content were recalculated to dry matter of material (see Table S2 of the Supplementary Material). Mass balances of the processes were calculated based on the weights and volumes used in each processing step (Table S2 of the Supplementary Material) and expressed as percentages of fungal DNA relative to the input barley, where the initial amount of fungal DNA was set to 100%.

### 4.4. Transcriptomics

RNA was extracted from 100 mg of frozen homogenised material using the TRIzol reagent (Invitrogen, Carlsbad, CA, USA) and purified with an RNeasy column in the presence of DNase (Qiagen, Hilden, Germany). RNA quality was assessed by agarose gel electrophoresis and the Agilent 4200 TapeStation System (Agilent Technologies, Santa Clara, CA, USA). Each sample was represented by three independent replicates. To ensure RNA integrity and quality for NGS analysis, a secondary check was performed at the company providing NGS analysis (Seqme, Prague, Czech Republic). Next, sequencing libraries were prepared for each biological replicate, targeting the 3’ end of the transcripts. Libraries were normalised to compensate for possible differences in RNA concentration and pooled for further analysis. Own NGS analysis was performed using the Illumina NovaSeq (Illumina, San Diego, CA, USA) platform with the following parameters: single-end sequencing, 50 base pairs per read, and a sequencing depth of 100 million reads. During the sequencing process, raw fastq files were generated for each sample. These files underwent data preprocessing to ensure quality and remove unwanted sequences. Initial quality assessment was conducted using FastQC (Babraham Bioinformatics, Cambridge, UK) and multiQC (MultiQC, Cambridge, UK) programs. Trim Galore (Babraham Bioinformatics, UK) was then employed to eliminate low-quality reads and adapter sequences. The resulting data were again evaluated using FastQC and multiQC. After preprocessing, the reads were mapped to the respective genome references using Hisat2 (Johns Hopkins University, Baltimore, MD, USA). The genome references were downloaded from Ensembl (https://fungi.ensembl.org/info/data/ftp/index.html, accessed on 11 June 2024) for individual *Fusarium* species.

Mapped reads were used for differential expression analysis. Mapped read counts were obtained with featureCounts and stored in a count matrix. Differential gene expression analysis was conducted using DESeq2 (Bioconductor, New York, NY, USA). Transcripts with a log2-fold change greater than 1 or less than −1 (indicating at least a 2-fold change in expression) and an adjusted *p*-value less than 0.05 were considered significantly differentially expressed. Pairwise comparisons were made between all samples to identify differentially expressed genes.

Gene annotation was based on identifiers from Ensembl for *Fusarium* species. For genes with incomplete or no annotation, cDNA sequences were retrieved from Ensembl and subjected to BLASTx against the UniRef90 database on the UniProt website (https://www.uniprot.org/blast, accessed on 11 June 2024). The best hits for individual searches were used as further annotation of the genes.

### 4.5. Analysis of Mycotoxins

**Analytical standards**: Certified standards of 57 mycotoxins with declared purities ranging from 96.5% to 100.0% were used for quantification. A detailed list of these standards is provided in Table S3 of the Supplementary Material. For calibration and spiking purposes, a composite mixture at a concentration of 1000 μg/L in acetonitrile was prepared and stored at –20 °C until use.

**Sample preparation**: All homogenised samples (in three biological replicates) were extracted using an internal validated method [47]. Briefly, the sample (equivalent to 2 g of dry matter; 1 g in the case of rootlets) was placed into a 50 mL centrifuge tube, and acidified water (0.2% formic acid (Merck, Darmstadt, Germany)) was added to a final volume of 10 mL. The mixture was shaken and left to steep for 30 min. Then, 10 mL of acetonitrile (LC-MS grade, Merck, Germany) were added, and the sample was extracted on a laboratory shaker at 240 min^−1^ for 30 min (IKA 240, IKA Labortechnik, Stuttgart, Germany). Anhydrous magnesium sulphate (4 g) and sodium chloride (1 g) (purity ≥ 98% and ≥99%, respectively, both obtained from Merck, Germany) were subsequently added The sample was vigorously hand-shaken for 1 min and then centrifuged at 10,000 min^−1^; 13,081× *g* (Rotina 380R, Hettich, Tuttlingen, Germany) for 5 min. The extract was transferred into a glass vial and stored at –20 °C prior to analysis. Each sample was processed in three repetitions.

**U-HPLC-HRMS/MS analysis**: The method was performed according to *Dzuman* et al. [47]. Chromatographic separation of target analytes was performed on an Acquity UPLC^®^ HSS T3 reverse phase column (100 mm × 2.1 mm, 1.8 μm; Waters, Milford, MA, USA) using the UltiMate^®^ 3000 U-HPLC system (Thermo Scientific, USA). Detection was performed using a Q-Exactive Plus^TM^ (Thermo Scientific, USA) high-resolution tandem mass spectrometer. An overview of the exact masses of target analytes, fragment ions, retention times, and analyte-specific normalised collision energies (NCE) is summarised in Table S4 of the Supplementary Material.

Mycotoxins were quantified using matrix-matched calibration with three calibration matrices—barley (for quantification of input barley, pre-soaked barley treated by PEF and without PEF treatment, steeped barley, green malt I and II); malt (quantification of malt); and rootlets (quantification of rootlets). The samples (barley, malt, and rootlets) used to prepare the external matrix-matched calibration series were free from mycotoxin contamination or contained only very low levels (these ‘blank’ samples have been available in the internal laboratory depositary and were collected during the continuous long-term research on mycotoxin). Calibration batches were prepared in the concentration range of 0.5–2500 μg/kg. The appropriate volume of the composite mixture (1000 μg/L) containing all mycotoxins was pipetted into the vials. The solvent (acetonitrile) was then evaporated under nitrogen, and the residue was reconstituted in ‘blank’ sample extract. The prepared calibration standards were stored at –20 °C until analysis. Recoveries were determined using artificially contaminated samples (‘spikes’) prepared in five replicates for each matrix at two concentration levels (60 and 500 μg/kg). Method performance characteristics are provided in Table S5 of the Supplementary Material. Mycotoxin concentrations were corrected for recovery and recalculated to dry matter of material (see Table S2 of the Supplementary Material). Mass balances of the processes were calculated by considering the weights and volumes used in particular processing steps (Table S2 of the Supplementary Material) and expressed as percentages, with 100% representing the initial mycotoxin content in the input barley.

### 4.6. Analysis of Malt Enzymatic Activity

Diastatic power was determined using methods approved by the EBC Analysis Committee [53] and expressed in Windisch-Kolbach units (WK u). The enzymatic activities of α-amylase and β-glucanase were determined using commercial Megazyme kits (α-Amylase Assay Kit—Ceralpha Method; β-Glucanase Assay Kit—Malt; and Microbial) according to the procedure recommended by the manufacturer [54,55] and expressed in units per kilogram (U/kg).

## Figures and Tables

**Table 1:** Fungal DNA and mycotoxin levels in input barley used for PEF experiments and malting.

	Species	Fungal DNA Content (µg/kg) (*n* = 9)		Produced Mycotoxins	Mycotoxin Content (µg/kg) (*n* = 9)	
		Mean ^a^	Range ^b^		Mean ^a^	Range ^b^
Barley grown in 2020				DON	896	781–1047
	*F. culmorum*	56	48–65	D3G	335	238–403
				3-acetylDON (3-ADON)	55	46–66
	*F. graminearum*			15-acetylDON (15-ADON)	7.3	6.4–8.7
		63	52–71	NIV	104	85–121
				ZEA	36	28–43
				Neosolaniol (NEO)	34	29–42
				Diacetoxyscirpenol (DAS)	4.2	3.4–5.2
	*F. sporototrichioides*	60	38–84	HT-2	542	486–627
				T-2	319	256–386
				Enniatin A (Enn-A)	8.1	4.9–14.4
				Enniatin A1 (Enn-A1)	42	27–57
	*F. poae*	81	63–98	Enn-B	373	313–458
				Enn-B1	138	104–172
				Beauvericin (BEA)	31	24–42
Barley grown in 2021	*F. culmorum*	15	12–18	DON	2094	1807–2347
				D3G	596	517–678
				3-ADON	62	50–71
	*F. graminearum*	8	5–13	15-ADON	17	12–22
				NIV	778	689–895
				ZEA	83	60–103
	*F. sporototrichioides*	48	34–58	NEO	12	5–17
				DAS	3.9	2.9–4.7
				HT-2	263	203–310
				T-2	85	60–97
				Enn-A	3.9	1.9–6.1
	*F. poae*	24	17–34	Enn-A1	21	14–29
				Enn-B	326	212–502
				Enn-B1	110	57–171
				BEA	50	40–62

^a^ Arithmetic mean of samples processed in the indicated number of replicates. ^b^ The variability between values within a repeated determination.

**Table 2:** Mycotoxin levels (µg/kg) in *Fusarium* cultures on Petri dishes used for inoculation of input barley.

*Fusarium* Species Strains	DON	D3G	3-ADON	15-ADON	NIV	ZEA	NEO	DAS	HT-2	T-2	Enn-A	Enn-A1	Enn-B	Enn-B1	BEA
*F. culmorum* B (VURV-F 425)	3503	<50	919	375	<50	20	34 ^a^	<2.0	22 ^a^	35 ^a^	<1.0	<1.0	<1.0	<1.0	<1.0
*F. graminearum* 12M1 (VURV-F 357)	<50	<50	<10	<5	<50	1.4	<1.0	<2.0	<2.0	<1.0	<1.0	<1.0	<1.0	<1.0	<1.0
*F. graminearum* 354 (VURV-F 354)	169	<50	29	10	<50	<1.0	21 ^a^	<2.0	43 ^b^	68 ^b^	<1.0	<1.0	<1.0	<1.0	<1.0
*F. graminearum* 52M1 (VURV-F 361)	<50	<50	<10	<5	<50	<1.0	<1.0	<2.0	132 ^b^	20 ^a^	<1.0	<1.0	<1.0	<1.0	<1.0
*F. poae* 5M (VURV-F 995)	<50	<50	<10	<5	151 ^a^	<1.0	1.3 ^a^	240	<2.0	3.2 ^a^	<1.0	<1.0	<1.0	<1.0	483
*F. poae* 80M1 (VURV-F 996)	<50	<50	<10	<5	342 ^b^	<1.0	5.8	24 ^b^	39	72	<1.0	<1.0	<1.0	<1.0	140
*F. sporotrichioides* 146 (VURV-F 146)	<50	<50	<10	<5	<50	5.2	35,376	1472	665,849	108,713	<1.0	<1.0	<1.0	<1.0	84
*F. sporotrichioides* 205 (VURV-F 205)	<50	<50	<10	<5	<50	<1.0	21,186	718	134,945	37,587	<1.0	<1.0	<1.0	<1.0	49
*F. sporotrichioides* 239 (VURV-F 239)	<50	<50	<10	<5	<50	<1.0	39,886	1549	716,174	128,106	<1.0	<1.0	<1.0	<1.0	96

^a^ Detected in one out of three replicates. ^b^ Detected in two out of three replicates.

**Table 3:** Malt enzyme activity results.

Enzyme	Unit	*Experiment I*		*Experiment II*	
		Control Malt	PEF-Supported Malt	Control Malt	PEF-Supported Malt
α-Amylase	U/kg	240	66	211	203
β-Glucanase	U/kg	603	80	448	503
Diastatic power	WK u.	364	202	251	255

**Table 4:** List of selected genes differentially expressed in at least one pairwise comparison of samples of interest, specifically: PEF-supported vs. control samples of green malt II, PEF-supported green malt II vs. input barley, and control green malt II vs. input barley. Log2 transformed changes (Log2 FC) in gene expression as recorded for respective pairwise comparisons of samples of interest are displayed. Up-regulated genes are highlighted in green, while down-regulated genes are highlighted in red. Genes with a significant change in expression (adjusted *p*-value < 0.05; Log2 FC ≥ 1 | ≤ −1) are marked with an asterisk (*). Descriptions of proteins that best match the cDNA sequences of selected genes as obtained from the UniRef90 database are provided.

		Log2FC			
Micromycete Species	Expressed Gene ID	PEF-Supported Green Malt II/Control Green Malt II	PEF-Supported Green Malt II/Input Barley	Control Green Malt II/Input Barley	Encoded Proteins Description
*F. culmorum*	FCUL_03776.1	2.401 *	3.527	1.293	Cell wall protein phiA
*F. culmorum*	FCUL_05985.1	2.234 *	4.348 *	2.269	Lysophospholipase
*F. culmorum*	FCUL_10667.1	0.735	2.555 *	1.879	Alcohol oxidase 1
*F. graminearum*	MDC_LOCUS513367	2.406 *	3.843 *	1.359	Cell wall protein phiA
*F. poae*	FPOA_06190	−1.473	3.601	5.128 *	ATP synthase
*F. poae*	FPOA_09175	−2.32	1.795	4.103 *	Endo-1,4-beta-xylanase C
*F. poae*	FPOA_13679	3.914	4.684 *	0.93	Cytochrome P450 monooxygenase
*F. sporotrichioides*	FSPOR_7594	−1.539	3.228	5.391 *	Ecp2 effector protein

**Table 5:** Primers and probes used for the quantification of individual *Fusarium* species DNA.

Name		Sequence (5′ → 3′)—Fluorochrom/Quencher	Reference
*F. culmorum*	Forward	TTCACTAGATCGTCCGGCAG	[49]
	Reverse	GAGCCCTCCAAGCGAGAAG	
	Probe	AAAGAAGTTGCAATGTTAGTG–VIC/MGB	
*F. gramineum*	Forward	CTCCGGATATGTTGCGTCAA	[50]
	Reverse	CGAAGCATATCCAGATCATCCA	
	Probe	TGAGAATGTCTTGAGGCAATGCGAACTTT–ABY/QSY	
*F. sporotrichoides*	Forward	GGTTGGCGTCTCACTTATAC	[51]
	Reverse	AATTTCTGATTCGCTAAAGTGG	
	Probe	CACACCCATAGTTACGTGTAA	
*F. poae*	Forward	GCTGAGGGTAAGCCGTCCTT	[50]
	Reverse	TCTGTCCCCCCTACCAAGCT	
	Probe	ATTTCCCCAACTTCGACTCTCCGAGGA–ABY/QSY	
*Fusarium* species (ITS)	Forward	AACTCCCAAACCCCTGTGAACATA	[52]
	Reverse	TTTAACGGCGTGGCCGC	
	Probe	CGCTCGAACAGGCATGCCCGCCAGAATAC–VIC/QSY	

## Data Availability

The original contributions presented in this study are included in the article/Supplementary Material. Further inquiries can be directed to the corresponding author(s).

## Supplementary Materials

The following supporting information can be downloaded at: https://www.mdpi.com/article/10.3390/toxins16120537/s1, Table S1: Results of BLASTx searching against the UniRef90 database for sequences of selected *Fusarium* genes. cDNA sequences of genes differentially expressed in selected pairwise comparisons of samples of interest were used, and top results for respective gene sequences are displayed. Figure S1: Photo of the PEF System (OMNIPEF, VITAVE, Czech Republic) (A) and the batch treatment chamber made from polymethylmetacrylate (Plexiglas) (B). Table S2: Mass balance during malting and brewing and analytical dry matter content used for mass balance calculations. Table S3: Overview of certified mycotoxin standards. Table S4: Overview of retention times, exact masses of m/z precursor ions, fragments of mycotoxins, and normalised collision energies (NCE). Precursor ions for fragmentation are highlighted. Table S5: Method performance characteristics, i.e., recovery, repeatability expressed as relative standard deviation (RSD) and limit of quantification (LOQ) (*n* = 5).
