# Supplementary material for: Application of Pulsed Electric Field During Malting: Impact on Fusarium Species Growth and Mycotoxin Production
Source: Toxins (Basel). 2024 Dec 12;16(12):537. doi: 10.3390/toxins16120537 (PMC11679037; doi:10.3390/toxins16120537)
Supplement: Supplementary file 1 [file toxins-16-00537-s001.zip › toxins-3320474-supplementary.pdf]

Supplementary material to:

Application of pulsed electric field during malting: Impact on *Fusarium* species growth and mycotoxin production

Nela Prusova[a], Marcel Karabin[b], Lukas Jelinek[b], Jana Chrpova[c], Jaroslava Ovesna[c], Pavel Svoboda[c], Tereza Dolezalova[a], Adam Behner[a], Jana Hajslova[a], Milena Stranska[a, *]

[a] *University of Chemistry and Technology Prague, Department of Food Analysis and Nutrition, Technicka 3, 166 28 Prague, Czech Republic*
[b] *University of Chemistry and Technology Prague, Department of Biotechnology, Technicka 5, 166 28 Prague, Czech Republic*
[c] *Crop Research Institute in Prague, Drnovska 507/73, 161 06 Prague, Czech Republic*

[*] **Corresponding author:**
Prof. Milena Stranska, Ph.D.
Email: milena.stranska@vscht.cz
Phone: +420 220 443 142

Table S1. Results of BLASTx searching against the UniRef90 database for sequences of selected *Fusarium* genes. cDNA sequences of genes differentially expressed in selected pairwise comparisons of samples of interest were used, and top results for respective gene sequences are displayed.

Gene ID[a]	Accession[b]	Description[c]	Organism[d]	Length[e]	Identities(%)[f]	E value[g]
**FCUL_03776.1**	UniRef90_K3V8M0	**Cell wall protein PhiA**	*Fusarium sambucinum* species complex	179	92.2	1.8E-116
**FCUL_05985.1**	UniRef90_A0A0E0S5J3	**Lysophospholipase**	*Fusarium sambucinum* species complex	653	88.7	0.0
**FCUL_10667.1**	UniRef90_UPI000023F5F6	**alcohol oxidase**	*Gibberella zeae** (strain ATCC MYA-4620 / CBS 123657 / FGSC 9075 / NRRL 31084 / PH-1)	671	99.8	0.0
**MDC_LOCUS513367**	UniRef90_K3UYP5	**Cell wall protein PhiA**	*Fusarium sambucinum* species complex	193	93.8	5.1E-131
**FPOA_06190**	UniRef90_A0A098DK15	**ATP synthase subunit beta**	Eukaryota	518	97.9	0.0
**FPOA_09175**	UniRef90_I1S3T9	**Endo-1,4-beta-xylanase C**	*Fusarium*	327	94.8	0.0
**FPOA_13679**	UniRef90_A0A2L2T747	**Cytochrome P450 monooxygenase**	*Fusarium sambucinum* species complex	530	89.1	0.0
**FSPOR_7594**	UniRef90_A0A2L2U037	**Ecp2 effector protein domain-containing protein**	*Fusarium sambucinum* species complex	153	94.2	1.8E-93

[a] Identifiers for genes of interest.
[b] UniRef accession number of the top BLASTx hit.
[c] Functional description of the protein associated with the best hit.
[d] Source organism of the protein in the best match.
[e] Length of the alignment between the translated query sequence (nucleotide sequence encoded by the gene of interest) and the matched protein sequence.
[f] Percentage of exact matches between the translated query sequence and the matched protein sequence.
[g] Statistical significance of the match, with lower values indicating higher significance.
* Synonym of *Fusarium graminearum*

jkbn

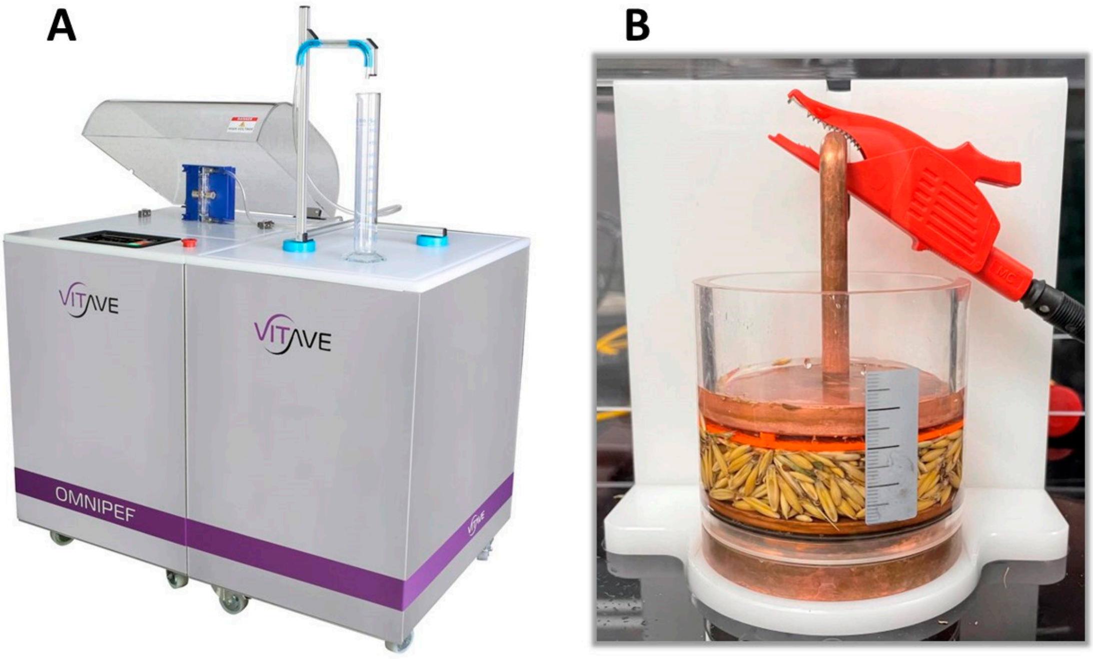

Figure S1. Photo of the PEF System (OMNIPEF, VITAVE, Czech Republic) (A) and the batch treatment chamber made from polymethylmetacrylate (Plexiglas) (B).

Table S2. Mass balance during malting and brewing and analytical dry matter content used for mass balance calculations.

Process		Matrix	Mass (kg)	Dry matter content (%)
Experiment I		Input barley	1.00	88
	Control	Steeped barley	1.65	58
		Green malt	1.31	59
		Malt	0.61	96
		Rootlets	0.03	96
	PEF-related	Steeped barley	1.68	55
		Green malt	1.35	53
		Malt	0.64	96
		Rootlets	0.04	96
Experiment II		Input barley	1.00	91
	Control	Pre-soaked barley without PEF treatment	1.22	74
		Steeped barley	1.55	51
		Green malt I	1.37	43
		Green malt II	1.11	42
		Malt	0.41	97
		Rootlets	0.03	97
	PEF-related	Pre-soaked barley treated by PEF	1.26	72
		Steeped barley	1.61	50
		Green malt I	1.45	48
		Green malt II	1.20	49
		Malt	0.43	96
		Rootlets	0.03	96

Table S3. Overview of certified mycotoxin standards.

No	Mycotoxin	CAS	Producer
1	15-Acetyldeoxynivalenol	88337-96-6	Romer Labs
2	3-Acetyldeoxynivalenol	50722-38-8	Romer Labs
3	Aflatoxin B1	1162-65-8	Merck
4	Aflatoxin B2	7220-81-7	Merck
5	Aflatoxin G1	1165-39-5	Merck
6	Aflatoxin G2	7241-98-7	Merck
7	Agroclavine	548-42-5	Romer Labs
8	Alternariol	641-38-3	Merck
9	Alternariol monomethyl ether	23452-05-3	Merck
10	Beauvericin	26048-05-5	Merck
11	Citrinin	518-75-2	Merck
12	Cyclopiazonic acid	18172-33-3	Merck
13	Deoxynivalenol	51481-10-8	Merck
14	Diacetoxyscirpenol	2270-40-8	Merck
15	Deoxynivalenol-3-glucoside	131180-21-7	Romer Labs
16	Enniatin A	2503-13-1	Merck
17	Enniatin A1	4530-21-6	Merck
18	Enniatin B	917-13-5	Merck
19	Enniatin B1	19914-20-6	Merck
20	Ergocornine	57432-60-7	Romer Labs
21	Ergocorninine	564-37-4	Romer Labs
22	Ergocristine	511-08-0	Romer Labs
23	Ergocristinine	511-07-9	Romer Labs
24	Ergocryptine	2706-66-3	Romer Labs
25	Ergocryptinine	511-10-4	Romer Labs
26	Ergometrine	60-79-7	Romer Labs
27	Ergosine	561-94-4	Romer Labs
28	Ergosinine	596-88-3	Romer Labs
29	Ergotamine	113-15-5	Romer Labs
30	Ergotaminine	639-81-6	Romer Labs
31	Fumonisin B1	116355-83-0	Merck
32	Fumonisin B2	116355-84-1	Merck
33	Fumonisin B3	136379-59-4	Romer Labs
34	Fusarenon X	23255-69-8	Merck
35	Gliotoxin	67-99-2	Merck
36	HT-2 toxin	26934-87-2	Romer Labs
37	Meleagrin	71751-77-4	Romer Labs
38	Mycophenolic acid	24280-93-1	Merck
39	Neosolaniol	36519-25-2	Merck
40	Nivalenol	23282-20-4	Romer Labs
41	Ochratoxin A	303-47-9	Merck
42	Patulin	149-29-1	Merck
43	Paxilline	57186-25-1	Merck
44	Penicillic acid	90-65-3	Merck
45	Penitrem A	12627-35-9	Merck
46	Phomopsin A	64925-80-0	Enzo Life Sciences
47	Roquefortine C	58735-64-1	Merck
48	Stachybotrylactam	163391-76-2	Romer Labs
49	Sterigmatocystin	10048-13-2	Merck
50	T-2 toxin	21259-20-1	Romer Labs
51	Tenuazonic acid	610-88-8	Romer Labs
52	Tentoxin	28540-82-1	Romer Labs
53	Verrucarol	2198-92-7	Merck
54	Verruculogen	12771-72-1	Romer Labs
55	Zearalenone	17924-92-4	Romer Labs
56	α-zearalenol	364-55-72-8	Merck
57	β-zearalenol	71030-11-0	Merck

Table S4. Overview of retention times, exact masses of m/z precursor ions, fragments of mycotoxins, and normalised collision energies (NCE). Precursor ions for fragmentation are highlighted.

No	Mycotoxin	Summary formula	RT (min)	ESI(−)		ESI(+)		NCE (%)	Fragment 1		Fragment 2	
				[M-H]$^-$	[M+CH_3COO]$^-$	[M+H]$^+$	[M+NH_4]$^+$		Summary formula	m/z	Summary formula	m/z
1	15-Acetyldeoxynivalenol	C17H22O7	2.69	337.1293	397.1504	339.1438	356.1704	10	[C17H23O7]$^+$	339.1438	[C17H21O6]$^+$	321.1333
2	3-Acetyldeoxynivalenol	C17H22O7	2.57	337.1293	397.1504	339.1438	356.1704	10	[C2H3O2]$^-$	59.0138	[C17H21O7]$^-$	337.1292
3	Aflatoxin B1	C17H12O6	3.45	311.0561	371.0772	313.0707	330.0972	60	[C14H9O4]$^+$	241.0495	[C15H9O5]$^+$	269.0444
4	Aflatoxin B2	C17H14O6	3.26	313.0718	373.0929	315.0863	332.1129	70	[C14H11O5]$^+$	259.0601	[C14H11O4]$^+$	243.0652
5	Aflatoxin G1	C17H12O7	3.01	327.0510	387.0722	329.0656	346.0921	60	[C13H11O3]$^+$	215.0703	[C14H11O4]$^+$	243.0652
6	Aflatoxin G2	C17H14O7	2.85	329.0667	389.0878	331.0812	348.1078	60	[C14H13O4]$^+$	245.0808	[C13H13O3]$^+$	217.0859
7	Agroclavine	C16H18N2	2.36	237.1397	297.1609	239.1543	256.1808	50	[C15H14N]$^+$	208.1121	[C12H11N2]$^+$	183.0917
8	Alternariol	C14H10O5	3.37	257.0455	317.0667	259.0601	276.0866	70	[C12H7O4]$^-$	215.0350	[C9H7O2]$^-$	147.0452
9	Alternariol monomethyl ether	C15H12O5	4.03	271.0612	331.0823	273.0758	290.1023	60	[C14H8O5]$^-$	256.0377	[C13H8O4]$^-$	228.0428
10	Beauvericin	C45H57N3O9	7.98	782.4022	842.4233	784.4168	801.4433	30	[C15H18O2N]$^+$	244.1332	[C9H12N]$^+$	134.0964
11	Citrinin	C13H14O5	2.87	249.0768	309.0980	251.0914	268.1179	50	[C13H13O4]$^-$	233.0808	[C13H15O5]$^-$	251.0914
12	Cyclopiazonic acid	C20H20N2O3	3.06	335.1401	395.1612	337.1547	354.1812	50	[C6H6O3N]$^-$	140.0353	[C11H8N]$^-$	154.0662
13	Deoxynivalenol	C15H20O6	2.07	295.1187	355.1398	297.1333	314.1598	10	[C2H3O2]$^-$	59.0139	[C15H19O6]$^-$	295.1187
14	Deoxynivalenol-3-glucoside	C21H30O11	1.98	457.1715	517.1927	459.1861	476.2126	20	[C20H27O10]$^-$	427.1610	[C21H29O11]$^-$	457.1715
15	Diacetoxyscirpenol	C19H26O7	3.53	365.1606	425.1817	367.1751	384.2017	20	[C17H23O5]$^+$	307.1540	[C15H17O2]$^+$	229.1223
16	Enniatin A	C36H63N3O9	8.36	680.4492	740.4703	682.4637	699.4903	20	[C12H20O2N]$^+$	210.1489	[C36H64O9N3]$^+$	682.4637
17	Enniatin A1	C35H61N3O9	8.20	666.4335	726.4546	668.4481	685.4746	20	[C35H62O9N3]$^+$	668.4481	[C12H20O2N]$^+$	210.1489
18	Enniatin B	C33H57N3O9	7.83	638.4022	698.4233	640.4168	657.4433	30	[C11H18O2N]$^+$	196.1332	[C11H20O3N]$^+$	214.1438
19	Enniatin B1	C34H59N3O9	8.03	652.4179	712.4390	654.4324	671.4590	30	[C34H60O9N3]$^+$	654.4324	[C11H18O2N]$^+$	196.1332
20	Ergocornine	C31H39N5O5	3.27	560.2878	620.3090	562.3024	579.3289	30	[C16H18ON3]$^+$	268.1444	[C15H15N2]$^+$	223.1230
21	Ergocorninine	C31H39N5O5	3.81	560.2878	620.3090	562.3024	579.3289	30	[C19H17O2N2]$^+$	305.1285	[C15H15N2]$^+$	223.1230
22	Ergocristine	C35H39N5O5	3.72	608.2878	668.3090	610.3024	627.3289	30	[C15H15N2]$^+$	223.1230	[C16H18N3O]$^+$	268.1444
23	Ergocristinine	C35H39N5O5	4.25	608.2878	668.3090	610.3024	627.3289	30	[C15H15N2]$^+$	223.1230	[C19H17O2N2]$^+$	305.1285
24	Ergocryptine	C32H41N5O5	3.67	574.3035	634.3246	576.3180	593.3446	30	[C16H18N3O]$^+$	268.1444	[C15H15N2]$^+$	223.1230
25	Ergocryptinine	C32H41N5O5	4.14	574.3035	634.3246	576.3180	593.3446	30	[C15H15N2]$^+$	223.1230	[C17H19N2O2]$^+$	283.1441
26	Ergometrine	C19H23N3O2	1.87	324.1718	384.1929	326.1863	343.2129	50	[C14H10NO]$^+$	208.0757	[C15H15N2]$^+$	223.1230
27	Ergosine	C30H37N5O5	3.01	546.2722	606.2933	548.2867	565.3133	30	[C15H15N2]$^+$	223.1230	[C16H18N3O]$^+$	268.1444
28	Ergosinine	C30H37N5O5	3.10	546.2722	606.2933	548.2867	565.3133	30	[C15H15N2]$^+$	223.1230	[C16H18N3O]$^+$	268.1444
29	Ergotamine	C33H35N5O5	3.15	580.2565	640.2777	582.2711	599.2976	30	[C15H15N2]$^+$	223.1230	[C17H17N2O3]$^+$	297.1234
30	Ergotaminine	C33H35N5O5	3.20	580.2565	640.2777	582.2711	599.2976	30	[C15H15N2]$^+$	223.1230	[C14H10NO]$^+$	208.0757
31	Fumonisin B1	C34H59NO15	4.37	720.3812	780.4023	722.3957	739.4223	30	[C22H40ON]$^+$	334.3104	[C22H44O3N]$^+$	370.3316
32	Fumonisin B2	C34H59NO14	5.46	704.3863	764.4074	706.4008	723.4274	30	[C22H42ON]$^+$	336.3261	[C22H40N]$^+$	318.3155
33	Fumonisin B3	C34H59NO14	4.99	704.3863	764.4074	706.4008	723.4274	30	[C22H42ON]$^+$	336.3261	[C22H44O2N]$^+$	354.3367
34	Fusarenon X	C17H22O8	2.23	353.1242	413.1453	355.1387	372.1653	10	[C2H3O2]$^-$	59.0139	[C17H21O8]$^-$	353.1242
35	Gliotoxin	C13H14N2O4S2	3.04	325.0322	385.0534	327.0468	344.0733	10	[C2H6O2NS]$^-$	108.0114	[C5H5O4NS]$^-$	174.9934
36	HT-2 toxin	C22H32O8	4.27	423.2024	483.2236	425.2170	442.2435	10	[C15H19O4]$^+$	263.1278	[C14H15O2]$^+$	215.1067
37	Meleagrin	C23H23N5O4	3.35	432.1677	492.1889	434.1823	451.2088	30	[C17H12O3N5]$^+$	334.0935	[C22H21O3N5]$^+$	403.1639
38	Mycophenolic acid	C17H20O6	4.68	319.1187	379.1398	321.1333	338.1598	50	[C11H11O4]$^+$	207.0652	[C10H9O3]$^+$	177.0546
39	Neosolaniol	C19H26O8	2.30	381.1555	441.1766	383.1700	400.1966	10	[C17H21O5]$^+$	305.1384	[C15H17O3]$^+$	245.1172
40	Nivalenol	C15H20O7	1.82	311.1136	371.1348	313.1282	330.1547	10	[C2H3O2]$^-$	59.0139	[C14H17O6]$^-$	281.1031

Table S4. Overview of retention times, exact masses of m/z precursor ions, fragments of mycotoxins, and normalised collision energies (NCE). Precursor ions for fragmentation are highlighted.

No	Mycotoxin	Summary formula	RT (min)	ESI(−)		ESI(+)		NCE (%)	Fragment 1		Fragment 2	
				$[M-H]^-$	$[M+CH_3COO]^-$	$[M+H]^+$	$[M+NH_4]^+$		Summary formula	m/z	Summary formula	m/z
41	Ochratoxin A	C20H18ClNO6	5.34	402.0750	462.0961	404.0895	421.1161	20	[C19H17O4NCl]$^+$	358.0841	[C11H8O4Cl]$^+$	239.0106
42	Patulin	C7H6O4	1.82	153.0193	213.0405	155.0339	172.0604	30	[C6H5O2]$^-$	109.0295	[C5H5O]$^-$	81.0346
43	Paxilline	C27H33NO4	6.93	434.2337	494.2548	436.2482	453.2748	20	[C27H32O3N]$^+$	418.2377	[C9H8N]$^+$	130.0651
44	Penicillic acid	C8H10O4	2.08	169.0506	229.0718	171.0652	188.0917	40	[C7H9O2]$^+$	125.0597	[C6H9O]$^+$	97.0648
45	Penitrem A	C37H44NO6Cl	4.42	632.2784	692.2996	634.2930	651.3195	40	[C32H33O5NCl]$^-$	546.2053	[C26H23O2NCl]$^-$	416.1423
46	Phomopsin A	C36H45ClN6O12	2.60	787.2711	847.2923	789.2857	806.3122	10	[C12H15O5N3]$^+$	281.1006	[C9H18O8N2]$^+$	282.1058
47	Roquefortine C	C22H23N5O2	4.10	388.1779	448.1990	390.1925	407.2190	40	[C8H9O2N4]$^+$	193.0720	[C17H16O2N5]$^+$	322.1299
48	Stachybotrylactam	C23H31NO4	6.31	384.2180	444.2392	386.2326	403.2591	60	[C9H8O3N]$^+$	178.0499	[C8H8O2N]$^+$	150.0550
49	Sterigmatocystin	C18H12O6	5.68	323.0561	383.0772	325.0707	342.0972	50	[C17H10O6]$^+$	310.0472	[C16H9O5]$^+$	281.0444
50	T-2 toxin	C24H34O9	4.89	465.2130	525.2341	467.2276	484.2541	10	[C21H31O7]$^+$	395.2064	[C12H17O4]$^+$	225.1121
51	Tentoxin	C22H30N4O4	4.52	413.2194	473.2406	415.2340	432.2605	30	[C18H22O2N3]$^+$	312.1707	[C9H19ON2]$^+$	171.1492
52	Tenuazonic acid	C10H15NO3	3.74	196.0979	256.1190	198.1125	215.1390	45	[C6H5O3]$^+$	125.0233	[C9H13O2]$^+$	153.0910
53	Verrucarol	C15H22O4	2.53	265.1445	325.1657	267.1591	284.1856	10	[C15H21O3]$^+$	249.1485	[C15H19O2]$^+$	231.1380
54	Verruculogen	C27H33N3O7	6.06	510.2246	570.2457	512.2391	529.2657	20	[C19H18O4N3]$^+$	352.1292	[C19H20O5N3]$^+$	370.1397
55	Zearalenone	C18H22O5	3.85	317.1394	377.1606	319.1540	336.1805	50	[C9H7O]$^-$	131.0502	[C10H7O3]$^-$	175.0401
56	α-zearalenol	C18H24O5	3.75	319.1551	379.1762	321.1697	338.1962	60	[C9H4O3]$^-$	160.0166	[C9H6O]$^-$	130.0424
57	β-zearalenol	C18H24O5	3.53	319.1551	379.1762	321.1697	338.1962	60	[C9H6O]$^-$	130.0424	[C9H4O3]$^-$	160.0166

Table S5. Method performance characteristics, i.e. recovery, repeatability expressed as relative standard deviation (RSD) and limit of quantification (LOQ) (n = 5).

No	Mycotoxin	Barley					Malt					Rootlets				
		Spike 60 µg/kg		Spike 500 µg/kg		LOQ (µg/kg)	Spike 60 µg/kg		Spike 500 µg/kg		LOQ (µg/kg)	Spike 60 µg/kg		Spike 500 µg/kg		LOQ (µg/kg)
		Recovery (%)	RSD (%)	Recovery (%)	RSD (%)		Recovery (%)	RSD (%)	Recovery (%)	RSD (%)		Recovery (%)	RSD (%)	Recovery (%)	RSD (%)	
1	15-Acetyldeoxynivalenol	92	4.8	95	9.9	10	89	5.3	92	3.1	10	86	6.7	89	4.2	25
2	3-Acetyldeoxynivalenol	99	3.0	107	2.4	2.5	94	3.8	102	3.6	5	94	3.6	102	3.1	10
3	Aflatoxin B1	99	2.1	93	1.9	0.5	99	2.3	93	2.0	0.5	82	2.5	77	1.7	0.5
4	Aflatoxin B2	95	0.9	89	1.6	0.5	91	1.3	85	1.1	0.5	86	1.9	83	6.5	0.5
5	Aflatoxin G1	94	2.8	92	2.3	0.5	89	3.3	87	2.9	0.5	82	3.0	80	2.4	0.5
6	Aflatoxin G2	102	1.8	100	2.2	0.5	101	2.2	99	1.8	0.5	85	2.4	83	3.1	1
7	Agroclavine	85	3.3	84	1.2	0.5	93	3.1	92	2.7	0.5	75	3.5	74	3.9	10
8	Alternariol	105	5.1	110	3.2	0.5	94	4.8	98	4.5	2.5	93	3.2	97	2.7	0.5
9	Alternariol monomethyl ether	110	2.8	101	2.0	0.5	108	3.1	99	3.6	0.5	101	4.6	93	3.4	0.5
10	Beauvericin	91	3.7	66	2.2	1	125	5.1	99	4.3	5	109	5.5	79	5.2	2.5
11	Citrinin	70	5.0	76	1.4	1	71	4.5	77	4.2	2.5	66	6.2	72	5.9	10
12	Cyclopiazonic acid	82	7.0	74	6.4	2.5	101	7.4	91	6.4	5	81	5.7	73	5.4	5
13	Deoxynivalenol	94	5.4	109	2.1	10	89	3.9	103	3.3	25	97	4.8	112	4.3	10
14	Deoxynivalenol-3-glucoside	28	10.9	44	2.4	25	27	8.7	43	7.6	50	25	8.1	39	7.6	25
15	Diacetoxyscirpenol	99	2.6	93	2.8	2.5	99	4.3	93	4.1	25	88	5.7	83	4.3	2.5
16	Enniatin A	91	2.5	96	4.1	0.5	95	3.4	100	3.0	1	85	4.6	90	3.9	0.5
17	Enniatin A1	90	2.4	92	2.5	0.5	92	2.9	94	2.4	5	80	3.7	82	4.2	1
18	Enniatin B	87	1.5	93	4.1	0.5	90	2.2	96	1.9	5	73	2.9	78	3.6	1
19	Enniatin B1	88	1.7	96	1.9	1	89	2.8	97	2.1	2.5	70	3.2	76	3.8	5
20	Ergocornine	87	4.7	99	1.5	2.5	82	5.1	93	4.7	2.5	92	5.5	105	5.2	5
21	Ergocorninine	96	1.2	97	2.7	1	98	1.7	99	1.5	2.5	108	2.9	109	3.7	1
22	Ergocristine	88	5.2	100	1.6	1	83	6.3	94	5.8	5	80	6.8	91	7.9	1
23	Ergocristinine	88	1.4	93	3.5	1	93	2.7	98	2.3	2.5	95	3.7	100	4.2	2.5
24	Ergocryptine	99	4.0	101	2.4	1	98	4.5	100	3.9	2.5	108	4.9	110	6.2	2.5
25	Ergocryptinine	98	2.0	98	3.1	1	100	2.9	100	2.2	2.5	113	3.6	113	3.2	2.5
26	Ergometrine	74	0.9	82	1.3	1	72	1.5	80	1.0	10	70	3.0	77	2.4	10
27	Ergosine	94	6.8	82	10.1	1	88	7.9	77	6.4	2.5	116	9.2	101	8.6	1
28	Ergosinine	104	7.2	91	2.0	1	115	8.4	101	5.4	2.5	113	8.1	99	7.6	10
29	Ergotamine	98	3.9	95	3.7	1	102	4.7	99	5.0	5	104	4.3	101	3.6	10
30	Ergotaminine	103	3.2	95	4.9	1	106	5.7	98	4.3	2.5	110	7.1	101	3.7	10
31	Fumonisin B1	148	4.9	79	4.1	25	148	5.6	79	6.2	50	149	5.9	97	4.5	50
32	Fumonisin B2	137	5.7	85	4.2	25	142	7.2	88	4.7	50	138	6.5	97	5.3	50
33	Fumonisin B3	142	4.6	91	3.7	25	142	5.2	91	4.7	50	145	4.9	93	5.1	50
34	Fusarenon X	110	5.1	87	4.1	10	116	4.8	92	5.1	25	128	5.6	101	4.9	50
35	Gliotoxin	135	4.5	71	3.0	10	154	3.7	81	6.9	50	-[1]	-[1]	93	4.8	100
36	HT-2 toxin	101	4.0	95	3.1	2.5	105	4.6	99	4.1	25	107	5.3	101	6.2	10
37	Meleagrin	100	4.2	93	2.3	0.5	85	5.3	79	6.3	2.5	110	6.7	102	5.8	5
38	Mycophenolic acid	93	5.1	92	4.6	1	100	6.2	99	5.7	5	84	5.6	83	4.3	2.5

Table S5. Method performance characteristics, i.e. recovery, repeatability expressed as relative standard deviation (RSD) and limit of quantification (LOQ) (n = 5).

No	Mycotoxin	Barley					Malt					Rootlets				
		Spike 60 µg/kg		Spike 500 µg/kg		LOQ (µg/kg)	Spike 60 µg/kg		Spike 500 µg/kg		LOQ (µg/kg)	Spike 60 µg/kg		Spike 500 µg/kg		LOQ (µg/kg)
		Recovery (%)	RSD (%)	Recovery (%)	RSD (%)		Recovery (%)	RSD (%)	Recovery (%)	RSD (%)		Recovery (%)	RSD (%)	Recovery (%)	RSD (%)	
39	Neosolaniol	103	4.4	91	5.0	0.5	112	4.9	99	3.8	5	102	7.3	90	6.4	5
40	Nivalenol	49	5.6	82	2.5	25	42	6.1	71	5.9	25	38	5.8	63	6.2	25
41	Ochratoxin A	102	2.9	94	2.3	5	106	3.7	98	4.8	25	100	4.9	92	5.3	5
42	Patulin	-[1]	-[1]	71	6.6	100	-[1]	-[1]	76	7.2	100	-[1]	-[1]	97	3.6	100
43	Paxilline	94	4.2	88	11.7	5	101	6.0	94	6.3	25	104	6.7	97	7.2	25
44	Penicillic acid	-[1]	-[1]	90	3.8	250	67	4.3	68	5.4	50	-[1]	-[1]	80	7.1	100
45	Penitrem A	96	2.5	93	2.0	5	103	3.2	100	4.6	5	100	3.1	97	4.6	2.5
46	Phomopsin A	-[1]	-[1]	80	1.7	100	-[1]	-[1]	89	5.2	100	-[1]	-[1]	94	4.8	250
47	Roquefortine C	96	2.1	88	1.6	1	98	3.3	90	3.8	10	87	3.8	80	4.7	1
48	Stachybotrylactam	79	6.8	88	3.0	5	83	7.2	93	6.7	5	82	7.0	91	6.4	5
49	Sterigmatocystin	96	2.7	100	1.1	0.5	88	3.9	92	3.3	2.5	84	4.5	88	4.2	0.5
50	T-2 toxin	102	4.1	94	4.7	0.5	103	4.3	95	4.5	0.5	103	4.3	95	5.7	0.5
51	Tentoxin	96	3.3	91	3.5	1	101	3.9	96	4.2	1	100	4.2	95	3.8	0.5
52	Tenuazonic acid	-[1]	-[1]	75	2.8	100	-[1]	-[1]	84	4.3	250	-[1]	-[1]	71	5.3	250
53	Verrucarol	105	8.4	101	6.1	50	-[1]	-[1]	86	3.7	250	98	6.4	94	5.1	50
54	Verruculogen	109	8.6	103	7.9	25	-[1]	-[1]	84	5.1	100	-[1]	-[1]	93	7.6	100
55	Zearalenone	96	1.9	104	2.2	0.5	93	2.3	101	3.7	1	88	2.6	95	3.3	0.5
56	α-zearalenol	90	1.9	89	2.0	2.5	89	3.5	88	2.9	2.5	93	3.9	92	4.6	2.5
57	β-zearalenol	103	3.0	91	2.3	2.5	111	4.1	98	3.4	2.5	108	4.4	95	3.7	2.5

[1] Recovery and RSD of these mycotoxins at the concentration level of 60 µg/kg could not be determined because the LOQ of these mycotoxins was higher than 60 µg/kg
